# Factors Associated With Walking Speed in Female Patients Attending Osteoporosis Outpatient Clinics: A Cross-Sectional Study of Sagittal Spinal Alignment, Osteoporotic Vertebral Fractures, and Handgrip Strength

**DOI:** 10.7759/cureus.114140

**Published:** 2026-08-07

**Authors:** Akira Kuwabara, Kazuhide Inage, Masaomi Yamashita, Sumihisa Orita, Yawara Eguchi, Yasuhiro Shiga, Masahiro Inoue, Miyako Suzuki-Narita, Takahisa Hishiya, Kohei Okuyama, Susumu Tashiro, Shuhei Ohyama, Noritaka Suzuki, Kosuke Takeda, Masaya Mizutani, Akihiro Iida, Yu Otake, Seiji Ohtori

**Affiliations:** 1 Department of Orthopaedic Surgery, Graduate School of Medicine, Chiba University, Chiba, JPN; 2 Department of Rehabilitation, Japan Community Health Care Organization (JCHO) Funabashi Central Hospital, Funabashi, JPN; 3 Department of Orthopaedic Surgery, Japan Community Health Care Organization (JCHO) Funabashi Central Hospital, Funabashi, JPN; 4 Department of Bioenvironmental Medicine, Graduate School of Medicine, Chiba University, Chiba, JPN

**Keywords:** handgrip strength, japanese rehabilitation, osteoporosis, osteoporotic vertebral fracture, sagittal vertical axis, walking speed

## Abstract

Objective

Walking speed is an important indicator of physical function and is associated with adverse outcomes such as falls and mortality. This study aimed to examine the associations between walking speed and sagittal spinal alignment, osteoporotic vertebral fracture (OVF), and handgrip strength in female patients aged 60 years or older attending osteoporosis outpatient clinics.

Methods

This exploratory cross-sectional study included 102 female patients aged ≥60 years with confirmed osteoporosis who attended osteoporosis outpatient clinics. Walking speed, sagittal vertical axis (SVA), OVF, handgrip strength, age, BMI, and lumbar bone mineral density (BMD) were assessed. Multiple linear regression analysis was performed using data from all 102 participants, with walking speed as the dependent variable.

Results

In the multivariable model, OVF (B = -0.1484, p < 0.001), SVA (B = -0.00307, p < 0.001), and handgrip strength (B = 0.0178, p = 0.002) were significantly associated with walking speed, whereas BMI, age, and lumbar BMD were not. The model had an R² of 0.478 and an adjusted R² of 0.446.

Conclusion

Walking speed in female patients aged 60 years or older attending osteoporosis outpatient clinics was associated with sagittal spinal alignment, OVF, and handgrip strength. These findings suggest that sagittal spinal alignment, vertebral fracture, and handgrip strength may be relevant factors to consider when interpreting reduced walking speed in this population.

## Introduction

Walking speed is a fundamental indicator of physical function that reflects mobility and has been associated with adverse outcomes such as falls, the need for long-term care, hospitalization, and death [1-3]. Clarifying the factors associated with reduced walking speed is therefore important for the early identification of functional decline and for understanding the clinical factors related to impaired walking function.

In addition to age-related declines in muscle strength and physical function, abnormal spinal posture and osteoporotic changes may contribute to reduced walking speed. In particular, poor sagittal spinal alignment may lead to forward trunk inclination and anterior displacement of the center of gravity, thereby adversely affecting standing balance and gait [4,5]. Osteoporotic vertebral fracture (OVF) has also been suggested to be associated with impaired physical function through spinal deformity, pain, reduced trunk function, and decreased activity levels [6,7]. Furthermore, handgrip strength is widely used as a simple indicator of overall muscle strength and frailty and has been reported to be associated with gait performance [8,9].

Previous studies have reported associations between walking speed and sagittal spinal alignment, OVF, and muscle strength. However, these factors have often been examined separately, and evidence regarding their relative associations with walking speed within the same osteoporosis outpatient population remains limited. Evaluating structural factors, such as sagittal alignment and vertebral fracture, together with a simple measure of muscle strength may provide a more integrated understanding of reduced walking speed in this clinical population. Therefore, the purpose of this exploratory cross-sectional study was to examine the associations between walking speed and sagittal vertical axis (SVA), OVF, and handgrip strength in female patients aged 60 years or older attending osteoporosis outpatient clinics after adjustment for age, BMI, and lumbar bone mineral density (BMD). We hypothesized that greater SVA, the presence of OVF, and lower handgrip strength would be associated with slower walking speed.

## Materials and methods

Study design

This was an exploratory cross-sectional study conducted at Chiba University Hospital, Chiba, Japan, and Japan Community Health Care Organization (JCHO) Funabashi Central Hospital, Funabashi, Japan, between April 2024 and March 2026. The study protocol was approved by the ethics review committee of the principal investigator’s affiliated institution (approval no. 2805). The study was conducted in accordance with the principles of the Declaration of Helsinki, and written informed consent was obtained from all participants.

Participants

The study population comprised female patients aged 60 years or older with a confirmed diagnosis of osteoporosis who attended osteoporosis outpatient clinics at the two institutions. Some patients had previously been diagnosed with osteoporosis, whereas others were referred because osteoporosis was suspected or low bone mineral density had been identified by dual-energy X-ray absorptiometry (DXA). In the latter group, osteoporosis was confirmed following evaluation at one of the participating institutions before study enrollment. Eligibility criteria included a confirmed diagnosis of osteoporosis before enrollment and the ability to understand the study explanation and provide informed consent independently. Exclusion criteria were spinal disorders with neurological abnormalities (including radiculopathy, motor paralysis, sensory deficits, diminished tendon reflexes, or clear imaging findings of neural compression), a history of surgery within the previous six months, and difficulty cooperating with the study assessments because of severe cognitive impairment or other reasons.

Between April 2024 and March 2026, 121 patients were assessed for eligibility. Of these, 10 were excluded because cognitive impairment made the assessments difficult, and nine were excluded because walking speed data were missing for four patients and handgrip strength data were missing for five patients. Consequently, 102 patients were included in the final analysis. The mean age of the participants was 73.7 ± 8.9 years.

Outcome measures

The variables assessed were 10-m walking speed, SVA, age, BMI, handgrip strength, the presence of OVF, and lumbar BMD. Age and BMI were obtained from medical records or information collected at the time of measurement.

10-m Walking Speed

The 10-m walking speed was measured as the comfortable walking speed during level-ground walking while wearing shoes. The timed section was 10 m, with 2-m acceleration and deceleration zones at either end. Participants were instructed to walk at their usual pace and began walking at the examiner’s signal. Measurements were performed twice, with sufficient rest between trials, and the mean value was used for analysis. None of the participants routinely used a walking aid in daily life.

Handgrip Strength

Handgrip strength was measured in the standing position using the same model of digital handgrip dynamometer at both institutions (Grip-D, T.K.K. 5401; Takei Scientific Instruments Co., Ltd., Japan). Before each use, the zero display was confirmed, and the devices were inspected according to the equipment management procedures at each institution. Two measurements were taken on each side, and the higher of the maximum values obtained from the right and left sides was used as the representative value for analysis. This method was adopted to provide a simple measure of maximal exertional capacity.

OVF

The presence of OVF was independently evaluated by two spine surgeons using plain thoracolumbar radiographs. Based on the concept of the semiquantitative method, the overall assessment incorporated morphological findings such as reduced vertebral height, wedge deformity, biconcave deformity, crush deformity, and endplate depression. In this study, OVF classification was not based on the strict application of the Genant semiquantitative method [10] but rather on a comprehensive clinical judgment integrating imaging findings and clinical information. Vertebral deformities without a clear traumatic history and with findings consistent with osteoporotic changes were classified as OVF. When necessary, CT or MRI findings were also reviewed, and both acute and old fractures were classified as OVF. Disagreements were resolved through discussion to reach a final judgment. To assess the reproducibility of OVF classification, Cohen’s kappa coefficient was calculated for inter-rater agreement based on the initial ratings.

SVA

**Figure 1:**
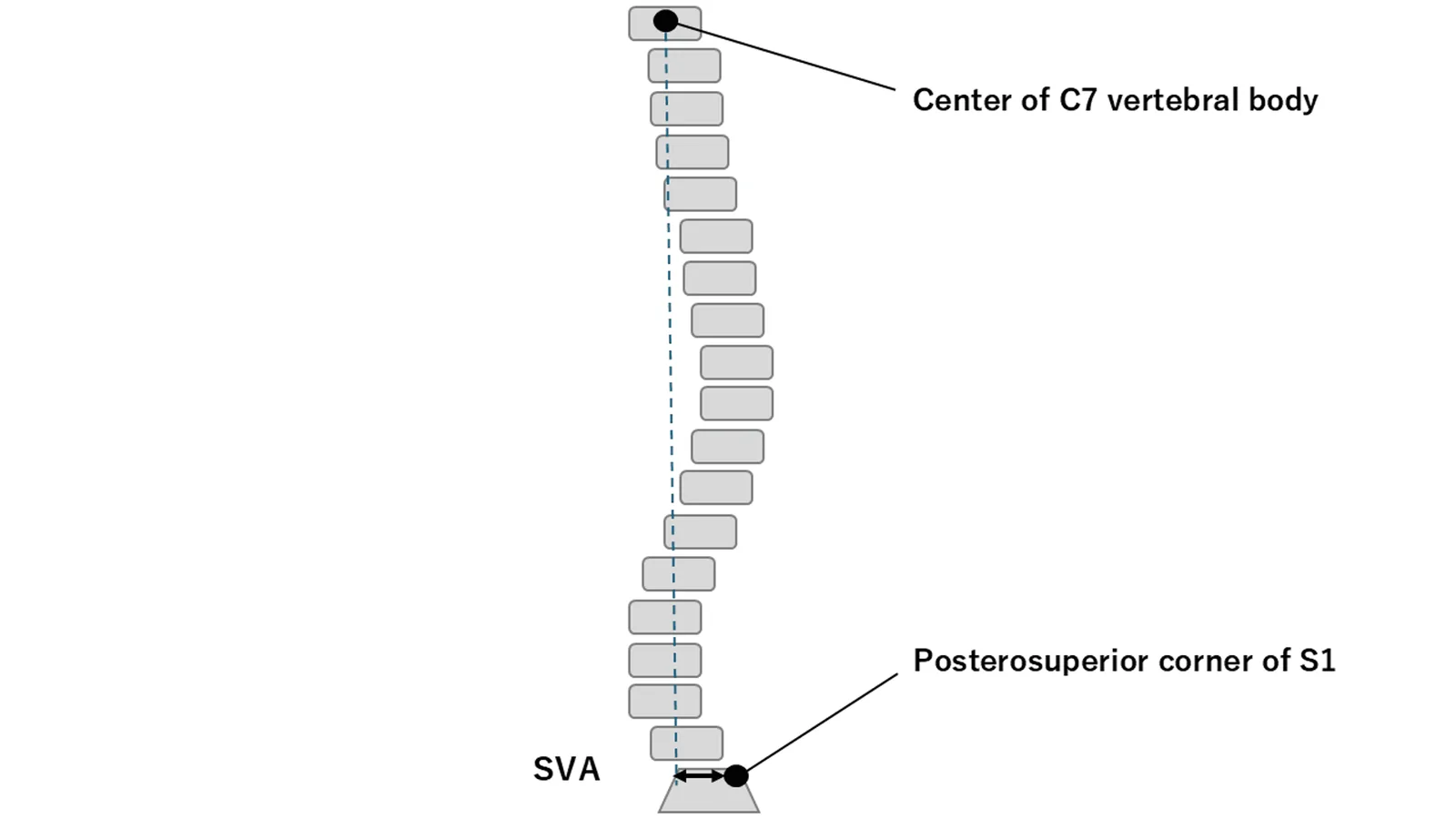
Schematic illustration of sagittal vertical axis (SVA) measurement. SVA was defined as the horizontal distance between the vertical plumb line dropped from the center of the C7 vertebral body and the posterosuperior corner of the S1 vertebral body. Image credit: Created by the authors using Microsoft PowerPoint for Microsoft 365 (Microsoft Corporation, Redmond, WA, USA).

SVA was evaluated as an index of sagittal spinal alignment using standing lateral whole-spine radiographs. SVA was defined as the horizontal distance between the vertical plumb line dropped from the center of the C7 vertebral body and the posterosuperior corner of S1 and was measured on the radiographs in millimeters (Figure 1).

Measurements were performed by one spine surgeon experienced in spinal radiographic assessment. In this study, a single representative measurement was used for each patient in the primary analysis. SVA measurements were performed according to predefined anatomical landmarks, and the measurement procedures were standardized across the two institutions.

Lumbar BMD

Lumbar BMD was measured using DXA, and the L1-L4 lumbar value was used for analysis. The measurement was expressed as a percentage of the young adult mean (%YAM). Vertebrae judged to affect the measurement, such as those with severe degenerative changes, vertebral fractures, postoperative changes, or calcification, were excluded. However, lumbar DXA values may still be influenced by degenerative changes and vertebral deformities and may not exclusively reflect bone fragility.

Standardization of measurement procedures

To minimize between-institution variability in the assessment procedures, data were collected only after standardizing the methods for measuring walking speed and handgrip strength, the standing conditions during imaging, and the basic procedures for image evaluation.

Statistical analysis

As a supplementary descriptive analysis to characterize participant characteristics, participants were categorized using the 50-mm SVA threshold employed in adult spinal deformity assessment [11,12]. SVA > 50 mm was operationally defined as positive sagittal malalignment, whereas SVA ≤ 50 mm served as the comparison category. However, because SVA is a continuous measure, it was analyzed as a continuous variable in the primary multiple regression analysis. Welch’s t-test was used to compare walking speed, age, BMI, handgrip strength, and lumbar spine BMD, and Pearson’s chi-square test was used to compare OVF prevalence. No inferential test was performed for participant counts or SVA because the groups were defined according to the prespecified SVA cutoff. For comparisons between the two institutions, Welch’s t-test was used for continuous variables, and Pearson’s chi-square test was used for OVF prevalence.

Multiple regression analysis was performed with walking speed as the dependent variable and BMI, lumbar BMD, OVF, SVA, handgrip strength, and age as independent variables. OVF was coded as 0 = absent and 1 = present. The regression analysis included all 102 participants. These independent variables were selected a priori based on previous studies and clinical relevance. Statistical significance was defined as p < 0.05, and continuous variables were presented as mean ± SD. All analyses were performed using JMP® 15 (SAS Institute Inc., Cary, NC, USA).

Before performing the multiple regression analysis, scatterplots of each continuous explanatory variable against walking speed were examined to assess linearity, and residual plots and normal Q-Q plots were used to assess the residual distribution and homoscedasticity. Correlations among the explanatory variables, multicollinearity, and the presence of influential points were also examined. To assess multicollinearity, the variance inflation factor (VIF) of each explanatory variable was examined; all VIF values were below 5, indicating no evident multicollinearity. These diagnostic assessments did not reveal any major deviations likely to materially affect the primary results.

This was an exploratory cross-sectional study, and no formal sample size calculation based on an a priori effect-size estimate was performed. However, because the planned multivariable regression model included six variables, the target sample size was set at approximately 100 participants, using a rough benchmark of at least 10 participants per variable to ensure stable estimation of the regression coefficients. Accordingly, the results of this study should be interpreted as hypothesis-generating.

Patients with missing values for variables required in the analyses were excluded before the final analytical cohort was established. The final dataset contained complete data for all variables included in the regression model. Because the missing data could not be assumed to have occurred completely at random, selection bias related to the exclusion of patients with missing data may remain. In addition, although the study was conducted at two institutions and the assessment procedures were standardized, differences in participant characteristics and measurement environments could not be completely eliminated.

## Results

Of the 121 patients screened, 10 were excluded because cognitive impairment made evaluation difficult, and nine were excluded because walking speed data were missing for four patients and handgrip strength data were missing for five patients, leaving 102 patients for the final analysis. Of the 102 participants included in the final analysis, 46 were recruited from Chiba University Hospital and 56 from JCHO Funabashi Central Hospital. Inter-rater agreement regarding the presence of OVF was κ = 0.72, indicating good agreement.

Among the 102 participants, 42 were classified into the SVA ≤ 50 mm group and 60 into the SVA > 50 mm group. In supplementary between-group comparisons, the SVA > 50 mm group had significantly slower walking speed, was older, and had a higher prevalence of OVF than the SVA ≤ 50 mm group. In contrast, no significant differences were observed in BMI, handgrip strength, or lumbar bone mineral density (Table 1).

**Table 1:** Participant characteristics overall and by SVA group. Data are presented as mean ± standard deviation or n (%). Continuous variables were compared using Welch’s t-test, and OVF prevalence was compared using Pearson’s chi-square test. Mean differences were calculated as the SVA > 50 mm group minus the SVA ≤ 50 mm group. The odds ratio for OVF represents the odds in the SVA > 50 mm group relative to the SVA ≤ 50 mm group. BMD: Bone mineral density; MD: Mean difference; OR: Odds ratio; OVF: Osteoporotic vertebral fracture; SVA: Sagittal vertical axis; YAM: Young adult mean.

Variable	Overall	SVA ≤ 50 mm	SVA > 50 mm	Test used	Test statistic	p-value	Effect estimate (95% CI)
Participants, n	102	42	60	Not applicable	-	N/A	N/A
Walking speed, m/s	0.72 ± 0.22	0.80 ± 0.13	0.66 ± 0.26	Welch’s t-test	t = 3.70	< 0.001	MD -0.14 (-0.22 to -0.06)
SVA, mm	60.4 ± 37.2	28.6 ± 18.7	82.6 ± 30.1	Not applicable	-	N/A	N/A
Age, years	73.7 ± 8.9	70.5 ± 8.7	76.1 ± 8.3	Welch’s t-test	t = -3.26	0.002	MD 5.60 (2.19 to 9.01)
BMI, kg/m²	20.8 ± 2.8	21.1 ± 3.0	20.7 ± 2.7	Welch’s t-test	t = 0.69	0.492	MD -0.40 (-1.55 to 0.75)
Handgrip strength, kg	19.5 ± 3.7	19.6 ± 3.7	19.4 ± 3.7	Welch’s t-test	t = 0.17	0.866	MD -0.20 (-1.68 to 1.28)
OVF, n (%)	32 (31.4)	4 (9.5)	28 (46.7)	Pearson’s chi-square test	χ² = 15.83	< 0.001	OR 8.31 (2.64 to 26.21)
Lumbar spine BMD (%YAM)	80.7 ± 15.9	82.7 ± 19.6	79.1 ± 12.5	Welch’s t-test	t = 1.05	0.299	MD -3.60 (-10.45 to 3.25)

Multiple regression analysis, with walking speed as the dependent variable, showed that OVF (B = -0.1484, p < 0.001), SVA (B = -0.00307, p < 0.001), and handgrip strength (B = 0.0178, p = 0.002) were significantly associated with walking speed. In contrast, BMI, lumbar bone mineral density, and age were not significantly associated with walking speed. The model explained 47.8% of the variance in walking speed (R² = 0.478; adjusted R² = 0.446; F(6, 95) = 14.52, p < 0.001) (Table 2). Among the standardized partial regression coefficients, SVA had the largest absolute value (β = -0.518), followed by OVF (β = -0.314) and handgrip strength (β = 0.299).

**Table 2:** Multiple linear regression analysis of walking speed. The dependent variable was walking speed, and the multiple regression analysis included all 102 participants. OVF was coded as 0 = absent and 1 = present. The 95% confidence intervals correspond to the unstandardized regression coefficients (B). Model statistics were R² = 0.478, adjusted R² = 0.446, F(6, 95) = 14.52, and p < 0.001. B: Unstandardized regression coefficient; BMD: Bone mineral density; OVF: Osteoporotic vertebral fracture; SE: Standard error; SVA: Sagittal vertical axis; β: Standardized partial regression coefficient.

Variable	B	Standard error	Standardized coefficient (β)	95% CI for B	t-value	p-value
Intercept	0.6313	0.2851	-	0.0653 to 1.1973	2.214	0.029
BMI	-0.01	0.0064	-0.127	-0.0228 to 0.0028	-1.551	0.124
Lumbar spine BMD (%YAM)	0.000714	0.00113	0.052	-0.001529 to 0.002957	0.632	0.529
OVF	-0.1484	0.0422	-0.314	-0.2322 to -0.0646	-3.515	< 0.001
SVA, mm	-0.003075	0.000482	-0.518	-0.004031 to -0.002118	-6.381	< 0.001
Handgrip strength, kg	0.01777	0.00547	0.299	0.00690 to 0.02863	3.246	0.002
Age, years	0.001596	0.002359	0.065	-0.003087 to 0.006279	0.677	0.5

Regarding multicollinearity, all explanatory variables had VIF values below 5, indicating no evident multicollinearity. Residual diagnostics and the assessment of influential points also revealed no major problems likely to materially affect the primary results.

A descriptive comparison based on the available background information for the 19 excluded patients showed that age, BMI, the proportion with OVF, and lumbar bone mineral density were generally similar to those of the analyzed group, although SVA tended to be higher in the excluded group (Table 3).

**Table 3:** Descriptive comparison of background characteristics between included and excluded participants. Data are presented as mean ± standard deviation or n (%). BMD: Bone mineral density; OVF: Osteoporotic vertebral fracture; SVA: Sagittal vertical axis; YAM: Young adult mean.

Variable	Included participants (n = 102)	Excluded participants (n = 19)
Age, years	73.7 ± 8.9	75.2 ± 6.4
BMI, kg/m²	20.8 ± 2.8	21.3 ± 3.9
OVF, n (%)	32 (31.4)	6 (31.6)
SVA, mm	60.4 ± 37.2	72.1 ± 28.3
Lumbar spine BMD (%YAM)	80.7 ± 15.9	78.1 ± 11.4

No statistically significant differences were observed between the two institutions in any of the demographic or clinical characteristics examined (Table 4).

**Table 4:** Comparison of participant characteristics between the two institutions. Data are presented as mean ± SD or n (%). Continuous variables were compared using Welch’s t-test, and OVF prevalence was compared using Pearson’s chi-square test. BMD: Bone mineral density; OVF: Osteoporotic vertebral fracture; SVA: Sagittal vertical axis; YAM: Young adult mean.

Variable	Chiba University Hospital (n = 46)	JCHO Funabashi Central Hospital (n = 56)	p-value
Age, years	73.3 ± 8.7	74.1 ± 9.1	0.652
BMI, kg/m²	21.2 ± 2.7	20.4 ± 2.9	0.153
Walking speed, m/s	0.70 ± 0.21	0.74 ± 0.23	0.361
Handgrip strength, kg	19.7 ± 3.6	19.3 ± 3.8	0.587
SVA, mm	59.8 ± 36.8	61.0 ± 37.6	0.871
Lumbar spine BMD (%YAM)	79.6 ± 15.6	81.8 ± 16.2	0.488
OVF, n (%)	15 (32.6)	17 (30.4)	0.807

## Discussion

In this study of female patients aged 60 years or older attending osteoporosis outpatient clinics, greater SVA, the presence of OVF, and lower handgrip strength were significantly associated with slower walking speed in the multivariable model. In contrast, BMI, lumbar BMD, and age were not significantly associated with walking speed in this model. These findings suggest that, in this study population, reduced walking speed may reflect multiple structural and functional factors, including sagittal spinal alignment, vertebral fracture, and handgrip strength.

The present findings should be interpreted in the context of previous studies that have individually reported associations between walking speed and sagittal spinal alignment, vertebral fracture, and muscle strength. The contribution of this study lies in the simultaneous evaluation of these structural and functional factors within the same osteoporosis outpatient population. This approach may help clarify how multiple clinically relevant factors coexist in relation to walking speed in this population, although the cross-sectional design of the present study does not allow conclusions regarding causality.

First, SVA was significantly associated with walking speed and had the largest absolute standardized partial regression coefficient among the variables included in the model. Greater SVA reflects greater anterior displacement of the trunk relative to the pelvis, which may increase postural control demands during walking.

Imagama S et al. reported that sagittal spinal alignment, muscle strength, and 10-m walking speed were related to balance and falls in middle-aged and older men [13]. Tokida R et al. also reported associations between sagittal spinal alignment and physical function in a community-based population of older Japanese adults [14]. The present study extends these findings by showing that greater SVA remained independently associated with slower walking speed after simultaneous adjustment for OVF, handgrip strength, age, BMI, and lumbar BMD in female osteoporosis outpatients. Compensatory changes in pelvic and lower-extremity posture may contribute to reduced gait efficiency or stability; however, gait patterns, compensatory movements, and postural sway were not directly assessed in this study, and the underlying mechanisms therefore cannot be determined. In the fitted multivariable model, a 10-mm greater SVA corresponded to an approximately 0.03 m/s lower walking speed. This finding supports sagittal alignment as a structural factor relevant to interpreting reduced walking speed in this population.

Second, OVF was significantly associated with walking speed even after adjustment for SVA and the other variables included in the multivariable model. This finding is consistent with the study by Arima K et al., in which women with two or more vertebral compression fractures had slower walking speeds than women without vertebral compression fractures [7]. Jacobs E et al. also reported altered spatiotemporal gait characteristics in patients with symptomatic osteoporotic vertebral compression fractures [15]. The present results add to these observations by demonstrating that the association between OVF and walking speed persisted even after adjustment for global sagittal alignment. This finding suggests that vertebral fracture may reflect aspects of reduced physical function that are not fully explained by sagittal malalignment alone. OVF may be associated with walking performance through several pathways, including pain, spinal deformity, reduced spinal mobility, and lower activity levels. However, because pain, physical activity, and other related factors were not directly assessed, the mechanisms underlying this association cannot be determined from the present study. In addition, OVF was evaluated only as present or absent; the number, location, severity, and chronicity of fractures were not examined. Therefore, further studies incorporating these fracture characteristics and relevant clinical factors are needed to clarify how OVF relates to walking speed.

Furthermore, lower handgrip strength was significantly associated with slower walking speed in the multivariable model after adjustment for the other variables included in the model. This result is consistent with previous studies showing that handgrip strength and walking speed represent related but distinct measures of physical function. Lee L et al. reported that both measures were useful for frailty screening in primary care [9], whereas Felix RJ et al. found that handgrip strength could help identify slow walking speed among geriatric outpatients [16]. The present study extends these findings to female osteoporosis outpatients and demonstrates that the association remained significant after adjustment for sagittal alignment and vertebral fracture. Handgrip strength is widely used as a simple indicator of overall muscle strength and physical reserve [8,9]. Walking speed is a composite measure influenced by multiple factors, including lower-extremity muscle strength, trunk function, balance ability, and neuromuscular coordination. Therefore, the observed association may reflect broader physical function rather than a direct effect of handgrip strength on walking performance. Handgrip strength may therefore provide complementary functional information alongside structural factors such as sagittal alignment and vertebral fracture. However, because handgrip strength does not directly assess lower-extremity or trunk extensor strength and detailed motor function was not evaluated in this study, this finding should be interpreted with caution.

Although participants in the SVA > 50 mm group were older than those in the SVA ≤ 50 mm group, age was not significantly associated with walking speed in the multivariable model. This finding should not be interpreted as indicating that age is unimportant for walking function. Because this cross-sectional analysis was not designed to clarify the pathways through which age may relate to walking speed and the sample size was limited, the absence of a significant association should be interpreted cautiously.

Similarly, BMI and lumbar BMD were not significantly associated with walking speed in the multivariable model. BMI reflects overall body size but does not distinguish between muscle mass and fat mass and may therefore have limited ability to capture physical characteristics directly related to walking performance. In addition, lumbar DXA measurements may be influenced by degenerative changes and vertebral deformity and may not fully reflect bone fragility. Although vertebrae considered unsuitable for measurement were excluded, such influences could not be completely eliminated. Therefore, the absence of significant associations between BMI and lumbar BMD and walking speed should be interpreted cautiously and does not exclude their potential relevance to physical function or osteoporosis-related health outcomes.

From a clinical perspective, the present findings suggest that when reduced walking speed is observed in female patients aged 60 years or older attending osteoporosis outpatient clinics, sagittal spinal alignment, vertebral fracture, and handgrip strength may be relevant factors to consider when interpreting it. In particular, the finding that both sagittal spinal alignment and vertebral fracture were associated with walking speed in the multivariable model suggests that these factors may represent distinct but complementary structural aspects to consider when evaluating the clinical background of reduced walking speed. However, because this was a cross-sectional study, these factors should not be regarded as direct intervention targets based on the present findings alone. These findings also do not imply that routine measurement of walking speed should be implemented for all osteoporosis outpatients.

This study has several limitations. First, because of its cross-sectional design, the direction of causality could not be determined. Second, the study included only 102 female patients aged 60 years or older who attended osteoporosis outpatient clinics at two institutions; therefore, caution is needed when generalizing the findings to men, community-dwelling older adults, or other outpatient populations. Third, important confounding factors such as pain, lower-extremity muscle strength, spinal mobility, balance ability, physical activity, and comorbidities were not assessed, leaving the possibility of residual confounding. In particular, knee and hip osteoarthritis were not systematically assessed either radiographically or based on symptoms. Because lower-extremity osteoarthritis may independently affect walking speed and may also influence the relationship between sagittal alignment and gait performance, its omission should be considered an important limitation of this study. Fourth, OVF was assessed only as present or absent, without consideration of fracture number, location, severity, or chronicity. In addition, although OVF classification was carefully performed based on imaging findings, the comprehensive clinical judgment involved may still have included some degree of subjectivity. Fifth, nine patients were excluded because of missing data before the final analytical cohort was established. Although the multiple regression analysis included all 102 participants in the final cohort, selection bias related to the exclusion of patients with missing data may remain. In the descriptive comparison between the 19 excluded patients and the analyzed group, the available background factors were generally similar; however, because SVA tended to be higher in the excluded group, it cannot be assumed that the exclusions occurred completely at random, and the magnitude of the associations may have been either underestimated or overestimated. Sixth, walking speed was assessed over a 10-m measurement section; therefore, the findings may not fully reflect sustained walking performance over longer distances. Seventh, although assessments were conducted at two institutions and the procedures were standardized, differences in measurement environments and participant characteristics could not be completely excluded.

## Conclusions

In female patients aged 60 years or older attending osteoporosis outpatient clinics, walking speed was associated with sagittal spinal alignment, OVF, and handgrip strength. These findings suggest that sagittal alignment, vertebral fracture, and handgrip strength may be relevant factors to consider when interpreting reduced walking speed in this population. However, because this was an exploratory cross-sectional study, the influence of unmeasured confounding, selection bias, and between-institution differences cannot be excluded, and causal interpretations should be made with caution. Future longitudinal studies are needed to clarify the temporal relationships between these factors and changes in walking function.
